# FDG-PET in HIV-Positive Patient with Extranodal Diffuse Large B-Cell Lymphoma

**DOI:** 10.1055/s-0044-1779751

**Published:** 2024-04-25

**Authors:** Faizullah Mashriqi, Graham Keir, Akarsh Vijayashankar, Joseph E. Glaser, Ana M. Franceschi

**Affiliations:** 1 Division of Neuroradiology, Department of Radiology, Northwell Health / Donald and Barbara Zucker School of Medicine, Lenox Hill Hospital, New York, New York, United States; 2 Division of Nuclear Medicine, Department of Radiology, Northwell Health / Donald and Barbara Zucker School of Medicine, Manhasset, New York, United States

**Keywords:** extranodal lymphoma, diffuse large B-cell lymphoma, perineural spread, PET/MRI, brain PET

## Abstract

Extranodal diffuse large B-cell lymphoma (DLBCL) is a heterogeneous disease process and an aggressive form of non-Hodgkin's lymphoma. We present a case of multiorgan involvement of DLBCL in a patient with documented risk factors, including [ ^18^ F] fluorodeoxyglucose positron emission tomography/magnetic resonance imaging findings highlighting striking perineural spread involving intracranial and extracranial segments of the bilateral trigeminal nerves.

## Case Report

**Fig. 1:**
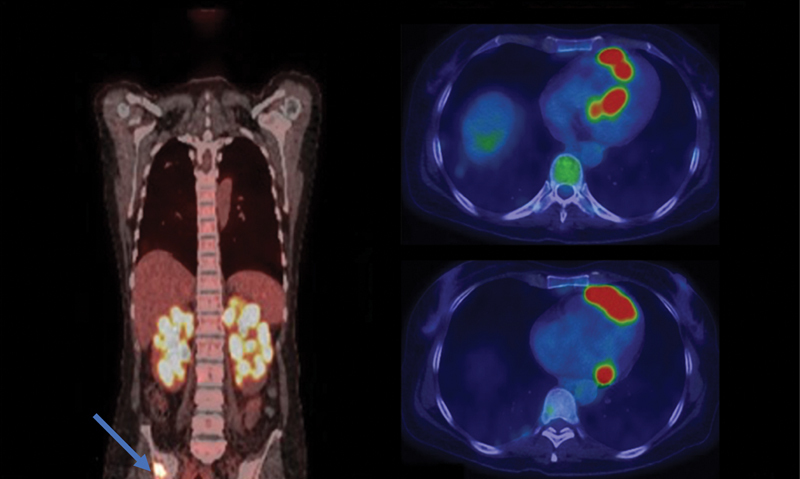
[ ^18^ F] fluorodeoxyglucose positron emission tomography/computed tomography ( ^18^ F-FDG PET/CT) from the skull base to mid-thigh (coronal, left and axial views, right) demonstrates avid radiotracer uptake in the renal parenchyma with a lobulated distribution and contour. Multiple skeletal FDG avid foci are present; a right iliac lesion is shown on coronal view ( *blue arrow* ). Multiple FDG avid myocardial foci are noted on axial views, with a discontinuous and irregular morphology suggesting malignancy.

**Fig. 2:**
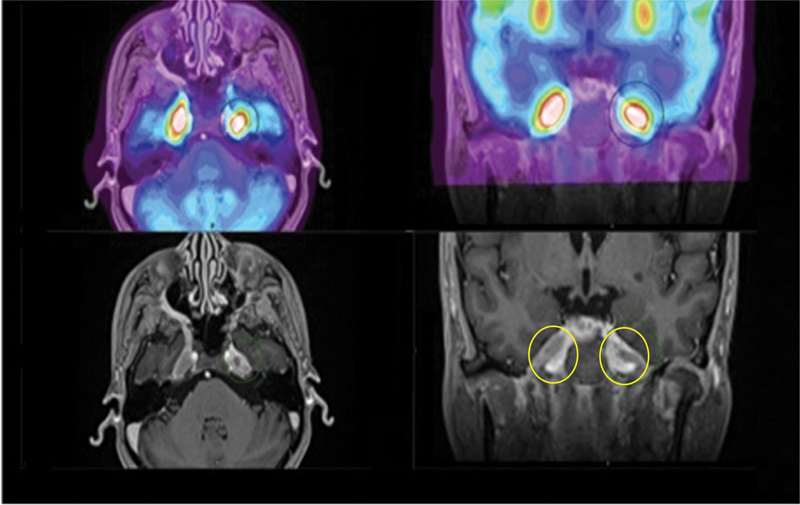
[ ^18^ F] fluorodeoxyglucose positron emission tomography/magnetic resonance imaging ( ^18^ F-FDG PET/MRI) of the brain (top, fused PET/MRI axial and coronal view; bottom, post-contrast T1-MPRAGE axial and coronal view) demonstrates bulky mass-like enhancement with corresponding avid tracer uptake involving the bilateral trigeminal nerves, including the cisternal segments, Meckel's cave, and extending extracranially along the V1-V3 branches consistent with holotrigeminal perineural spread of disease. This is best visualized on the bottom right image where perineural spread of tumor extends through expanded foramen ovale along the bilateral V3 divisions, yellow circles. MPRAGE, magnetization-prepared rapid acquisition gradient echo.

**Fig. 3:**
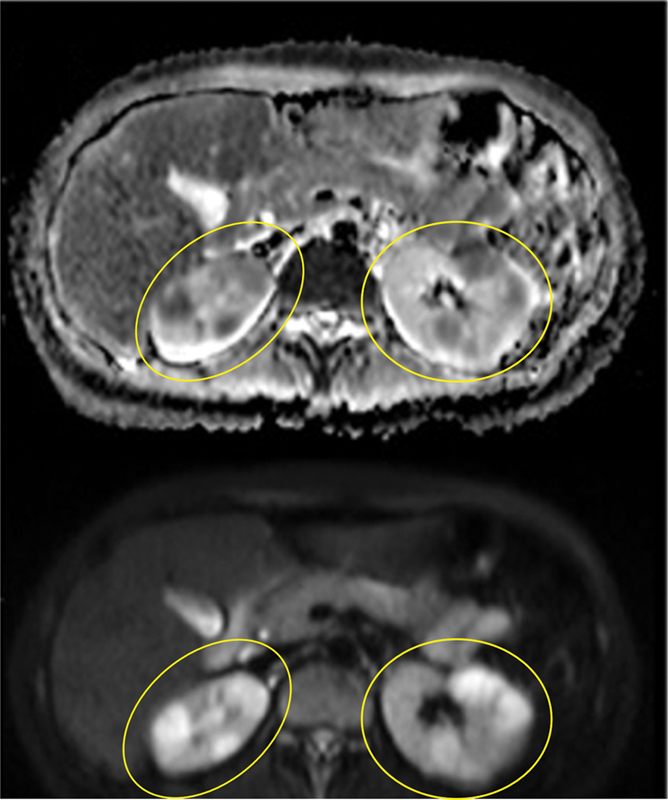
Abdominal magnetic resonance imaging with apparent diffusion coefficient map ( *top* ) and diffusion-weighted imaging ( *bottom* ) at the level of the kidneys shows bilateral diffusion restricting renal lesions, yellow circles.

**Fig. 4:**
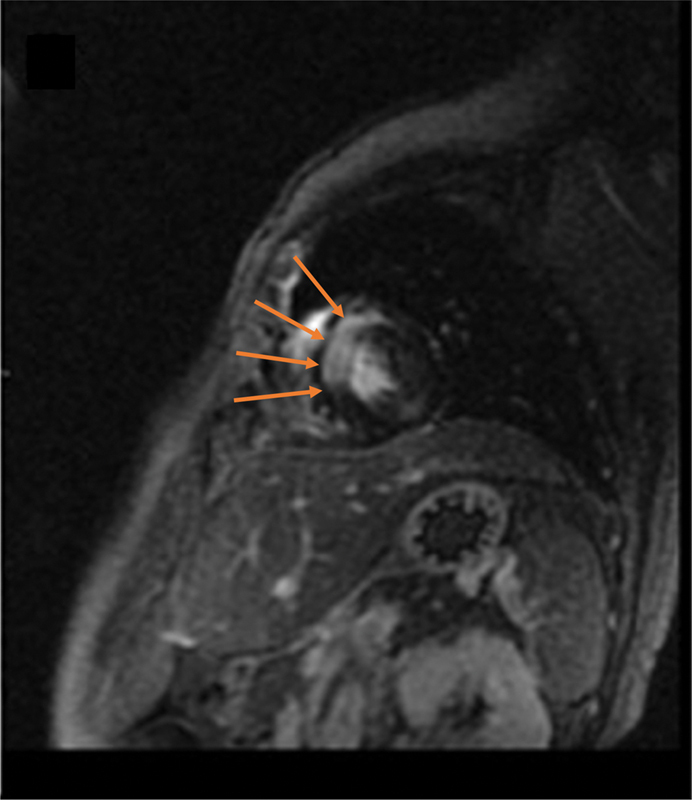
Cardiac magnetic resonance imaging demonstrates late gadolinium mural enhancement of the interventricular septum ( *orange arrows* ), which correlated with avid myocardial uptake on [ ^18^ F] fluorodeoxyglucose positron emission tomography/computed tomography.

A 42-year-old female presented to the emergency department with severe left facial pain and numbness. Initial evaluation did not reveal the etiology and the patient was discharged with outpatient follow-up. She returned 2 months later with persistent symptoms and was admitted for a thorough workup. Neuroaxis magnetic resonance imaging (MRI) revealed bilateral trigeminal nerve lesions and renal lesions on spine imaging. Whole body [ ^18^ F] fluorodeoxyglucose positron emission tomography/computed tomography ( ^18^ F-FDG PET/CT) demonstrated multiple hypermetabolic foci in the lungs, kidneys, myocardium, skeleton, and stomach ( Fig. 1 ). Brain ^18^ F-FDG PET/MRI ( Fig. 2 ) showed bulky mass-like enhancement with corresponding avid tracer uptake involving the bilateral trigeminal nerves, including the cisternal segments, Meckel's cave, and extending extracranially along the V1-V3 branches consistent with holotrigeminal perineural spread of disease. Abdominal MRI ( Fig. 3 ) demonstrated bilateral diffusion restricting renal lesions corresponding to hypermetabolic foci on FDG-PET. Lastly, cardiac MRI ( Fig. 4 ) demonstrated late gadolinium mural enhancement of the interventricular septum that correlated with avid myocardial uptake on FDG-PET. Renal biopsies were obtained and consistent with diffuse large B-cell lymphoma (DLBCL). Serology workup demonstrated human immunodeficiency virus-1 (HIV-1) with a CD4 count of 87 and a viral load of 3.5 million. Antiretroviral therapy and chemotherapy were initiated.

## Discussion

There are an estimated 150,000 new cases of DLBCL annually, accounting for 30% of non-Hodgkin's lymphoma with a median age in the mid-60s. 1 DLBCL is a heterogenous disease process that can arise from an extranodal locality in up to one-third of cases. 2 Common extranodal locations include skin and soft tissues, bone, and the gastrointestinal and genitourinary tracts. Renal involvement is associated with a very poor prognosis and indicates stage IV disease in over 90% of cases. 3 4 Case reports suggest that patients with renal lymphoma have an increased risk of central nervous system (CNS) dissemination, especially those with bilateral or bulky renal lesions. 5 Additionally, in a study of 2,164 patients with newly diagnosed aggressive B-cell lymphoma (80% DLBCL), involvement of the kidneys and/or adrenal involvement was determined to be an independent risk factor for CNS recurrence. 6

While most cases of DLBCL arise de novo without a history of lymphoma, low-grade B-cell lymphomas can transform into DLBCL. Multiple known risk factors for DLBCL have been identified including genetic susceptibility, viral exposure (HIV, Epstein-Barr virus, human herpesvirus 8, hepatitis C virus, and hepatitis B virus), immunodeficiency, as well as environmental and occupational exposures. DLBCL initially presents extranodally in approximately 40% of cases according to a population-based study of 1,575 patients. 7 The diagnosis is often missed as the symptoms may be similar to other inflammatory diseases. 8 Immunosuppression is a well-documented risk factor for the development of DLBCL. In fact, approximately 70% of all non-Hodgkin's lymphomas in HIV-infected patients are DLBCL. 9 In the era of antiretroviral therapy, survival of HIV-associated DLBCL approaches 75% and these outcomes following R-CHOP (rituximab, cyclophosphamide, daunorubicin, vincristine, prednisolone) regimen are similar to HIV negative patients. 10

The critical role of ^18^ F-FDG PET in the workup of patient with DLBCL has been previously characterized. Notably, ^18^ F-FDG PET/CT is more likely to upstage aggressive lymphoma when compared with CT alone, given that subcentimeter nodes and extranodal sites of disease are not readily apparent on structural imaging, but are markedly avid and readily identifiable by FDG-PET. Baseline PET/CT is typically recommended to obtain accurate staging of DLBCL at presentation. Furthermore, patients with poor response to treatment or early relapse may undergo stem cell transplantation, in which case interpretation of post-therapy ^18^ F-FDG PET often relies heavily on a baseline scan. 11 While MRI has been described as the gold standard for identifying perineural tumor spread, 12 our case report highlights the added value of hybrid ^18^ F-FDG PET/MRI.

When encountered with multifocal extranodal FDG avidity, particularly in immunosuppressed patients, it is critical to include opportunistic infections in the differential diagnosis. In the CNS, opportunistic infections may include progressive multifocal leukoencephalopathy, cytomegalovirus, cryptococcus, aspergillus, tuberculosis, and neurosyphilis, among others. 13 While most of these infections have presentations different than the perineural spread observed in our patient, neurosyphilis and aspergillus infections have been described as mimickers of perineural tumor spread in the literature. 14

This case demonstrates multiorgan involvement of DLBCL in a patient with documented risk factors. There have only been a few reported cases of extranodal involvement of the trigeminal nerve, usually unilaterally 15 or bilaterally in cases of recurrent lymphoma, 16 with our case involving the bilateral intracranial and extracranial segments of the trigeminal nerves at diagnosis. It is vital for neuroradiologists to be familiar with both frequent and rare sites of involvement, given that HIV+ patients may present with extranodal involvement in unusual locations.
